# Postural orthostatic tachycardia syndrome: A conundrum for patients and healthcare providers

**DOI:** 10.1097/01.NPR.0000802968.90212.70

**Published:** 2021-12-23

**Authors:** Holly M. Cline, Adam Einhardt

**Affiliations:** **Holly M. Cline** is a Master of Nursing student at Carson Newman University, Jefferson City, Tenn.; **Adam Einhardt** is an assistant professor of graduate nursing at Carson Newman University, Jefferson City, Tenn.

**Keywords:** autonomic nervous system, irritable heart, presyncope, postural orthostatic tachycardia syndrome, soldier's heart, syncope, tachycardia

## Abstract

Postural orthostatic tachycardia syndrome is an underdiagnosed disorder of the autonomic nervous system. The median time to achieve correct diagnosis is 2 years and may take more than 10 years for some patients. Symptoms can be devastating to the daily life of patients and can result in long-term disability. Treatment availability is limited due to the need for further studies.

**Figure:**
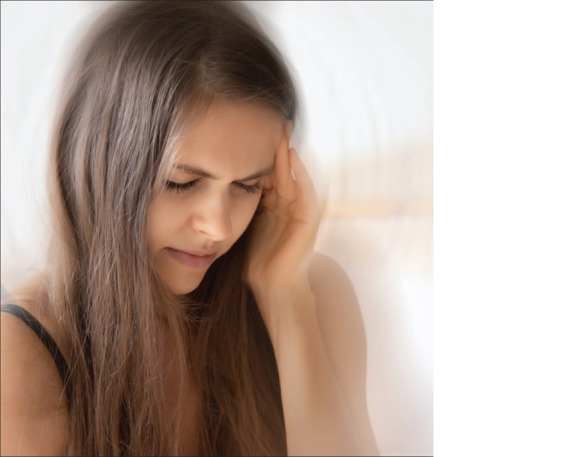
No caption available.

Postural orthostatic tachycardia syndrome (POTS) is a chronic, debilitating disorder of the autonomic nervous system that is widely underdiagnosed by healthcare professionals and has a devastating impact on the quality of patients' daily lives.^1^,^2^ The incidence of POTS in developed countries varies from 0.2% to 1% with up to 3 million cases in the US alone.^1^,^3^ POTS significantly impacts females 13 to 50 years of age; its prevalence ratio in females-to-males is 5:1.^1^,^2^ While POTS can occur in individuals of any race or ethnicity, a survey of patients with POTS found that 93% were White.^4^

POTS was first recognized in 1871 by Jacob Mendes Da Costa, who referred to the condition as “soldier's heart” or “irritable heart.” However, it was not formally recognized until 1993 by Dr. Ronald Schondorf and Dr. Philip Low.^1^

POTS is defined in the 2015 Heart Rhythm Society (HRS) expert consensus statement as the presence of symptoms of autonomic dysfunction upon standing, which may manifest as lightheadedness, fatigue, palpitations, nausea, blurred vision and/or tremors, with a sustained increase in heart rate of 30 beats/minute or more (40 beats/minute or more for individuals ages 12-19) within 10 minutes of standing from a supine position and without orthostatic hypotension.^1^,^5^-^7^ The Canadian Cardiovascular Society (CCS) position statement on POTS further delineates that while sustained orthostatic hypotension within 3 minutes of standing precludes a diagnosis of POTS, transient initial orthostatic hypotension lasting less than a minute may occur in some patients with POTS.^7^

Patients may have presyncope or syncope, with presyncope being more common.^3^ The symptoms of tachycardia and presyncope must be present in the absence of other causes of orthostatic tachycardia, such as thyroid dysfunction, dehydration, anemia, or the use of medications that affect autonomic regulation.^8^ The median time from initial presentation to diagnosis is 2 years, with about 15% of patients not receiving the diagnosis for over 10 years.^4^,^8^,^9^ The fragmentation of healthcare, episodic events, and decreased provider knowledge and familiarity with POTS all contribute to underdiagnosis of the condition. This lag in diagnosis, along with potentially debilitating symptoms of POTS, can contribute to or increase depression and anxiety. The disability resulting from symptoms of POTS has been compared with that of rheumatoid arthritis, end-stage renal disease, congestive heart failure, and chronic obstructive pulmonary disease.^3^,^9^,^10^ Although symptoms can be debilitating, life expectancy seems to be unaffected.^3^,^10^ Studies have found that certain comorbidities frequently occur with POTS, such as hypermobile Ehlers-Danlos syndrome, irritable bowel syndrome, fibromyalgia, chronic fatigue syndrome, migraines, and autoimmune diseases.^4^,^6^,^11^

POTS is known to be triggered by viral and bacterial infections, such as influenza and Epstein-Barr virus.^12^ In addition, there are case reports of individuals developing symptoms of and fulfilling the diagnostic criteria for POTS 6 to 8 months following recovery from COVID-19 infection.^13^ It has been suggested that the risk of developing POTS post-COVID-19 is higher in those with a preexisting, underlying autoimmune and/or inflammatory process or in those with a history of concussion.^12^

## Physiology of standing

Upon changing positions from supine to standing, there is an instantaneous shift of blood from the thorax to the lower extremities and the splanchnic circulation.^6^ A secondary shift of about 10% to 25% of plasma volume occurs from the vasculature to the interstitial space due to gravitational forces.^6^ The changes in position result in impairment of cardiac filling and decreased BP and stroke volume.^6^ The heart rate and systemic vascular resistance increase. The autonomic nervous system is responsible for counteracting hemodynamic changes resulting from fluid shifts. Baroreceptors stimulate the sympathetic nervous system and are located within the walls of the aortic arch and carotid sinus. These baroreceptors provide a negative feedback loop for heart rate and BP and cause constriction of blood vessels, which increases vascular resistance and escalates blood return to the heart.^6^ While the baroreceptors stimulate the sympathetic nervous system, they simultaneously suppress the parasympathetic nervous system.^6^ These compensatory mechanisms are capable of maintaining homeostasis within a 5 mm Hg increase in diastolic BP and a 10 to 20 beats/minute increase in heart rate.^6^ Alterations within the pathways have the potential to cause tachycardia and/or hypotension.

## Pathophysiology of POTS

The pathophysiology of POTS is unknown and is still under investigation through various studies. Although the exact cause of POTS is unknown at this time, autoimmunity, hypovolemia, genetics, and a dysregulation of the autonomic nervous system are all thought to play a role in the syndrome.^11^

### Mechanisms

Various mechanisms have been implicated in the pathophysiology of POTS which are not mutually exclusive and which often overlap.^5^,^14^

Neuropathic mechanisms may play a pathophysiologic role in approximately one half of patients diagnosed with POTS and are thought to be due to partial autonomic denervation and failure of the vasculature of the lower extremities to maintain resistance while in an upright position.^14^,^15^ This causes the blood to pool in the lower extremities, resulting in BP instability and tachycardia and is thought to have an autoimmune etiology, which is the result of the formation of autoantibodies against alpha3-acetylcholine receptors of the peripheral ganglia.^8^,^15^

Studies have found that autoimmunity plays a role in POTS, but the exact mechanism is unknown. Autoantibodies against muscarinic receptors M1 to M3 have been found in over 87% of patients with POTS.^14^ Other autoantibodies found in patients with POTS target cardiac lipid raft-associated proteins, adrenergic G-protein coupled receptors, alpha-1-adrenergic receptors, and beta-1- and beta-2-adrenergic receptors.^14^

Hyperadrenergic stimulation in POTS is thought to be genetic. This manifestation affects up to 50% of patients with POTS and is considered to involve a centrally driven sympathetic activation characterized by supine vasoconstriction and tachycardia.^5^,^8^,^14^,^15^ These patients have high norepinephrine levels (600 pg/mL or greater) when in the upright position.^5^,^14^ In some cases, this may be due to the impaired clearance of neurotransmitters at the synaptic cleft due to the deficiency of norepinephrine transporters (NET).^8^ Some common antidepressants and attention-deficit disorder medications may induce NET deficiency, leading to this hyperadrenergic state.^14^ Patients with hyperadrenergic POTS frequently present with episodes of tachycardia, hypotension, tachypnea, hyperhidrosis, palpitations, anxiety, and tremulousness.^15^

Mast cell activation syndrome may be present in some patients with POTS.^14^ These patients experience severe episodic flushing with sinus tachycardia.^14^ The patient will have a hyperadrenergic response to position change and an elevated urine methylhistamine during flushing episodes.^14^

Hypovolemia occurs in approximately 70% of patients with POTS.^14^ The average plasma volume deficit is about 13%.^14^ It is suspected that the fluid deficit is associated with an impaired renin-angiotensin-aldosterone system which lowers sodium retention.^14^

## Symptoms

The broad range of symptoms in POTS makes it difficult to identify, leading to a prolonged length of time from symptom onset to correct diagnosis.^3^ It has been noted that, in some cases, precipitating events such as an illness, surgery, pregnancy, or a traumatic incident occurred within 3 months prior to symptom onset.^4^

Symptoms of sympathetic hyperactivity can include tachycardia, palpitations, chest pain/discomfort, and tremors.^3^ Cerebral hypoperfusion may occur with POTS and results in blurred vision, headaches, lightheadedness, cognitive difficulties, and generalized weakness.^3^ Nonspecific complaints can include sleep disturbances, fatigue, pain, and affective symptoms.^16^ Patients often have presyncope and may occasionally have neurally-mediated syncope, which includes situational syncope, carotid sinus syndrome, and neurocardiogenic syncope.^17^ Symptoms of POTS also often include bowel/bladder dysfunction such as diarrhea; constipation; urinary frequency, urgency, and retention; and vascular phenomena such as blood pooling in the lower extremities.^16^ Gastrointestinal (GI) symptoms are common in patients with POTS. Commonly reported GI symptoms include nausea (86%), irregular bowel movements (71%), abdominal pain (70%), constipation (70%), heartburn (64%), and bloating (59%).^15^ This is thought to be due to an impairment in the sensorimotor function of the autonomic nerves within the gut.^15^ The alterations in motility and function of the GI tract in POTS can result in dehydration and nutritional consequences such as decreased nutrient absorption. Patients may have restricted eating habits—not related to eating disorders—resulting in issues such as folate and vitamin B12 deficiencies, which may cause megaloblastic anemia or even neurologic disorders, or underweight.^15^

### BMI decrease in POTS

Limited studies have suggested that the body mass index (BMI) of patients with POTS may be decreased. A study of 52 patients with POTS between the ages of 14 and 29 years showed decreased BMI in patients with reduced peripheral blood flow.^18^,^19^ Patients with POTS who have reduced peripheral blood flow also have an abnormally high level of angiotensin II, which is correlated with lower BMI in these patients.^18^,^19^ Increased angiotensin II within the CNS can produce sympatho-excitation, which is a major factor in the subgroup of hyperadrenergic POTS.^19^

## Diagnosis

Due to the extensive range of symptoms and variable levels of disability in POTS, it is very important that the healthcare provider obtain an accurate and thorough history and physical exam.

### History

Activity level and exercise information should be obtained.^9^ Some patients may present after an illness that required a lengthy period of bed rest leading to deconditioning. Patients may also experience a decrease in food and fluid intake, resulting in dehydration. Dietary history, including daily water and sodium intake, will assist in ruling out the possibility of dehydration as the cause of presyncope or syncope. Information on use of prescription medications, over-the-counter medications, and/or illegal drugs that may contribute to symptoms should also be obtained. A family history of heart disease, joint hypermobility, autoimmune disease, and/or neurologic disease should be collected.^9^,^15^ A thorough investigation of the details of the patient's symptoms and any occurrences of syncope or presyncope should be pursued, such as duration, frequency, triggers, associated symptoms, and impact on function.^6^ A comprehensive review of systems should be conducted with special attention to the cardiac, respiratory, gastrointestinal, neurologic, and urinary systems. Screening for depression, anxiety, and suicidal ideation should be conducted given the increased risk of suicide in this population.^9^

**Table:** Diagnostic criteria for POTS^5^,^7^

**Symptoms of autonomic dysfunction with standing**
• Lightheadedness
• Fatigue
• Palpitations
• Nausea
• Blurred vision
• Tremors
**+**
**Autonomic function testing**
• Sustained increase in heart rate of at least 30 bpm above baseline within 10 minutes of standing (for individuals age 20+ years)
or
• Sustained increase in heart rate of at least 40 bpm above baseline within 10 minutes of standing (for individuals ages 12 to 19 years)
**+**
**In absence of**
• Sustained orthostatic hypotension (decrease in systolic BP of more than 20 mm Hg or in diastolic BP of more than 10 mm Hg) within 3 minutes of standing

### Physical assessment

The physical exam should include all systems with an extensive assessment of the cardiac and neurologic systems due to the high incidence of hyperadrenergic signs and symptoms. These systems should be assessed for tachycardia, palpitations, arrythmias, and pallor. Extremities should be assessed for discoloration due to pooling of blood in the lower extremities, which gives the extremities a bluish/purple, mottled color.^9^ The extremities should also be assessed for joint hypermobility, temperature, swelling, weakness, tremors, excessive sweating, and pulse strength.

### Testing

Various testing modalities are used to rule out other disorders and diseases and to test the autonomic nervous system for dysfunction. Testing consists of blood tests, urine tests, ECGs, and autonomic function tests. There are no specific labs available to definitively identify POTS.

***Labs***. Serum studies are performed to rule out other possible causes of symptoms and disease. Tests to perform are a complete blood count (CBC) to aid in the identification of underlying anemia, cancers, and chronic infections; comprehensive metabolic panel (CMP), to evaluate kidney and liver disease and electrolyte imbalance; thyroid panel, to rule out thyroid disease; morning serum or salivary cortisol to evaluate for adrenal insufficiency; and plasma catecholamines, such as norepinephrine, and their metabolites, which can be measured during tilt table testing described below.^20^

A 24-hour urine sample measuring catecholamines and sodium levels should be ordered.

***Cardiac monitoring***. An ECG should be obtained to assess for tachycardia or arrythmias. A 24-hour Holter monitor should be worn by the patient to assess for arrythmias, along with 24-hour ambulatory BP monitoring.^21^

***Autonomic function testing***. Assessment for orthostatic tachycardia can be obtained by measuring the patient's heart rate and BP in a supine position followed by a standing position at 1-, 3-, 5-, and 10-minute intervals.^9^,^21^ Patients who have POTS will experience symptoms of autonomic dysfunction and the heart rate will increase by at least 30 beats/minute (or by at least 40 beats/minute in patients 12 to 19 years old) without an accompanying drop in BP.

The gold standard to diagnose POTS remains the tilt table test, which assesses changes in heart rate and BP along with symptoms due to positional changes.^20^,^22^ BP and heart rate are obtained at baseline and then throughout the test. The patient is strapped to a table, which tilts the patient into different positions throughout the test and then measures the patient's physical and hemodynamic response to positional changes.

## Treatment

Treatment for POTS focuses on minimizing symptoms, as there is currently no cure. Given the difficulty often associated with the treatment of POTS, upon diagnosis, a multidisciplinary approach should be pursued, which may include specialists in cardiology, neurology, psychology, physical therapy, physiatry, and others as needed.^5^,^15^ Statements on the condition from the HRS and CCS are available, as noted earlier.^5^,^7^ Current treatments consist of both nonpharmacologic and pharmacologic methods.

### Nonpharmacologic interventions

Patients with hypovolemia should increase their daily fluid and salt intake to increase the fluid volume within the intravascular space and assist in preventing dehydration. Two to three liters of water and 10 to 12 g of sodium daily is recommended by the HRS for patients with POTS and can be accomplished through diet or supplemental sodium tablets.^5^,^7^,^23^ The CCS position statement recommends oral fluid intake of at least 3 L daily and sodium intake of 10 g daily.^7^ Medications, supplements, food, or drinks that may decrease fluid volume—such as caffeine, tea, and diuretics—or worsen tachycardia or associated symptoms should be reviewed and potentially discontinued or avoided.^14^ Wearing waist-high compression stockings with 30-40 mm Hg of pressure assists in decreasing blood pooling in the lower extremities and directs blood flow more centrally.^14^

Short-term exercise of at least 30 minutes every other day for 3 months was found to decrease heart rate and increase baroreflex sensitivity in patients with POTS.^22^ It is important that the patient start a graduated exercise plan, beginning with recumbent exercises, and take caution not to overexert.^14^ The patient should avoid activities that may cause overheating. Patients should also take safety precautions to prevent injury to themselves. If the patient feels faint while walking or standing, they should sit or lay down immediately and elevate their legs above heart level. This is to avoid injury or head trauma, which could result from falling from a standing position. Elevating the legs above heart level assists in redirecting blood flow from the extremities back to the heart.

Practicing a regular sleep pattern will assist in addressing disrupted circadian rhythms.

Any mental health concerns should be addressed. Referral for psychological counseling may be of benefit to patients with POTS due to the sudden and unexpected changes in lifestyle that may result in depression or anxiety. Patients who have comorbid mental health disorders, including suicidal ideation, should be referred as appropriate based on the situation, such as to mental health providers, the ED, or crisis intervention services.

### Pharmacologic interventions

Limited studies have shown a few medications to effectively treat some symptoms of POTS. Prescription medications should be reserved for use in patients with severe symptoms who do not respond to nonpharmacologic treatment.^14^ The use of these medications for treatment of POTS or the symptoms of POTS is off-label as they have not been evaluated by the FDA for this purpose. Medications used in the management of POTS typically address tachycardia, peripheral vasoconstriction, or sympathetic tone, but fail to address chronic fatigue, cognitive dysfunction, gastrointestinal symptoms, and insomnia.^9^,^14^

The CCS position statement on POTS states that the most commonly used medications for the management of POTS include propranolol, midodrine, pyridostigmine, fludrocortisone, ivabradine, clonidine, methyldopa, and occasional 0.9% sodium chloride I.V. infusions.^7^ All are off-label for the treatment of POTS in the US. A review of studies and literature has suggested that the most effective and useful medications in the treatment of POTS may be certain beta-blockers, selective serotonin reuptake inhibitors (SSRIs), and fludrocortisone.^2^

Low-dose propranolol is suggested for improvement of symptoms related to sinus tachycardia.^5^ Beta-blockers should be titrated and used cautiously due to the possibility of increasing symptoms of fatigue.^2^

The literature review identified several studies supporting the effectiveness of SSRIs for management of POTS.^2^ However, there are no recommendations for their use in the HRS consensus statement or in the CCS position statement. Of note, one study included in the literature review suggested use of the serotonin-norepinephrine reuptake inhibitor (SNRI) venlafaxine; however, the HRS recommends against this.^2^,^5^ If SNRIs are used, close monitoring should occur due to the possible adverse reactions of increased norepinephrine levels in some patients with POTS.^2^

The mineralocorticoid fludrocortisone may be considered for plasma volume expansion and the sensitization of the peripheral adrenergic receptors to catecholamines.^2^ It is recommended to avoid fludrocortisone in patients with a history of migraines due to the possibility of exacerbation and increase in symptoms.^21^ There is also the possibility of edema and hypokalemia and early-onset osteoporosis in young women taking this medication.^7^

Midodrine is recommended for patients with a tendency to have hypotension.^7^,^23^ Low-quality evidence suggest that pyridostigmine may be useful, in combination with a beta-blocker, in improving orthostatic tachycardia.^7^ Several studies have suggested that ivabradine may be an option for patients who are intolerant of beta-blockers and have significant symptomatic orthostatic tachycardia.^7^ Clonidine is recommended for patients with hyperadrenergic symptoms such as palpitations, tremors, or significant orthostatic hypertension.^7^

A 1-2 L 0.9% sodium chloride bolus administered I.V. over 1-2 hours is recommended as second-line rescue therapy. This intervention should only be used in the event of occasional short-term decompensation or limited other situations. Routine administration of I.V. fluids is not recommended, although the position statement clarifies that it may be acceptable in certain extreme cases involving chronic vomiting or diarrhea, severe gastroparesis, or limited oral fluid intake due to comorbidities to prevent chronic hypovolemia.^7^

Other more invasive procedures have been performed but are not recommended as treatment for POTS or its symptoms. For example, studies have shown that ablation of the sinus node has not demonstrated efficacy and may worsen symptoms.^7^ Jugular venoplasty, also known as “liberation treatment,” is the balloon dilation and stenting of the superior jugular vein. While it has been performed for presumed chronic cerebrospinal venous insufficiency, it may cause harm and hasn't demonstrated efficacy; thus, the CCS recommends against its use in the treatment of POTS.^7^

## Quality of life and disability

The symptoms of POTS are debilitating and can lead to decreased quality of life and disability. Due to the severity of symptoms, about a third of patients diagnosed with POTS are unable to continue educational or work activities.^24^ A study completed by Barbic et al. found that patients with POTS who were working had a moderate to severe burden of autonomic symptoms and a concomitant reduced work ability compared with healthy individuals.^24^ Symptoms reported in this study likely impact the ability to perform both physical activities (including prolonged standing) and mental tasks and included dizziness, presyncope, light-headedness, vision disturbances, and difficulty concentrating. This study also revealed that symptoms may be exacerbated by work environments that exposed patients to excessive heat, intensive physical exertion, and stressful conditions.^24^

## Mental health and increased suicide risk

Chronic illness and disability have devastating effects on patients' mental health, putting them at increased risk for depression, anxiety, and suicide. The severity of symptoms can be life-altering, reducing the patient's ability to work, attend school, and function in normal everyday activities. It is reported that 50% of patients with POTS are at high risk of suicide with 15% to 18% reporting past suicide attempts and 13% reporting another likely attempt.^10^ Many patients have expressed their frustration with healthcare providers who imply that the condition is “all in their heads.”^25^ This leaves the patient feeling lost and hopeless and can further increase depression and heighten the risk of suicide.

## Conclusion

POTS' broad array of symptoms has life-altering consequences for the patients it affects. The lack of definitive testing and provider knowledge of the syndrome can lead to misdiagnosis and/or direct inappropriate blame toward the patient. Increased awareness and education of POTS is needed in the healthcare community to properly diagnose and effectively treat patients in a timely manner. Further investigations and studies are needed to determine specific pathology of the syndrome, develop more efficient and effective treatment measures, and assist patients in improving their daily lives.
